# Lumefantrine quantification on pre-treated filter paper dried blood spots for clinical field trials

**DOI:** 10.1186/s12936-026-06048-y

**Published:** 2026-07-20

**Authors:** Daniel Blessborn, Natpapat Kaewkhao, Joel Tarning

**Affiliations:** 1 Mahidol Oxford Tropical Medicine Research Unit, Faculty of Tropical Medicine, Mahidol University, 8Th Floor, 50Th Anniversary Chalermprakiat Building, 420/6 Rajvithi Rd., Bangkok, 10400 Thailand; 2 Centre for Tropical Medicine & Global Health, Nuffield Department of Medicine, University of Oxford, Oxford, UK

**Keywords:** Lumefantrine, Dried blood spots, Filter paper, Malaria

## Abstract

Artemether-lumefantrine is the most widely used antimalarial treatment for uncomplicated Plasmodium falciparum malaria. Malaria predominantly affects populations in rural areas of tropical and subtropical regions. Drug resistance is a growing concern, and monitoring antimalarial efficacy remains crucial for detecting changes in treatment performance and tracking potential resistance trends. In this study, we present an efficient method for collecting blood samples in resource-limited settings, using dried blood spots on pre-treated filter paper, which enhances the stability of lumefantrine. This approach builds on previous methods by incorporating rapid sample processing through semi-automated punching robots for 96-well plate formats, quick sample cleanup via filtration through a phospholipid removal plate, and expedited analysis using liquid chromatography-tandem mass spectrometry (LC–MS/MS) with a turnaround time of just 2 min per sample. The method also achieves a significantly improved the lower limit of quantification of 15 ng of lumefantrine per ml of blood (i.e. LLOQ of 15 ng/ml). A Phenomenex Luna-CN 50 × 2 mm, 3 µm LC column was used, with a flow rate of 400 µl/min and a mobile phase consisting of acetonitrile and ammonium formate buffer (10 mM, 0.5% formic acid) in a 67:33 (v/v) ratio. The linear range for quantification spanned from 15 to 15,000 ng/ml, based on three 3.2 mm punched discs from a 50 µl blood spot. Accuracy was within ± 15% of the validation criteria, and precision was demonstrated by intra-assay variability of less than 7.2% and inter-assay variability of less than 12% across all concentration levels. The stability of lumefantrine in dried blood spots on pre-treated filter paper was demonstrated using a sample set obtained from another research group, which had been stored in the dark under low-humidity conditions for up to 16 years. The method showed no endogenous interference or interference from the concomitant drug artemether and is suitable for use in clinical trials.

**Supplementary Information:**

The online version contains supplementary material available at https://doi.org/10.1186/s12936-026-06048-y.

## Introduction

Malaria remains a major public health threat in tropical and subtropical regions, with an estimated 282 million cases and 610,000 deaths in 2024 [1, 2]. It is caused by the *Plasmodium* parasite, which infects red blood cells and is transmitted by *Anopheles* mosquitoes [1]. Artemether-lumefantrine is the first-line treatment for uncomplicated *P. falciparum* malaria in most countries [2, 3].

Artemether is a fast-acting drug with a short elimination half-life of just 2 h, resulting in a rapid reduction in parasite load during the three days of drug administration. In contrast, lumefantrine has a longer half-life of 100–120 h and eliminates remaining parasites not cleared by artemether, thereby reducing the risk of malaria recrudescence [4]. Lumefantrine is highly bound to plasma proteins (99.7%), and plasma is the most commonly used matrix for quantification in pharmacokinetic studies [5]. Following the standard 6-dose regimen (twice daily for 3 days), plasma concentrations reach 5.6–12.6 µg/ml and decrease to about 643 ng/ml after 7 days of treatment initiation [4, 6]. Plasma lumefantrine concentrations are well-correlated between venous and capillary samples, though lumefantrine concentration in capillary samples are typically 5–15% lower [7, 8]. Gallay et al. found a strong correlation between lumefantrine concentrations in plasma and dried blood spot (DBS) samples, with a blood-to-plasma ratio of 0.56, indicating that blood concentrations are about half those in plasma [9].

Drug resistance is a growing concern, and monitoring antimalarial efficacy is critical to track resistance and to update treatment policies [1]. Most therapeutic efficacy studies still show > 95% cure rates for artemether-lumefantrine in Africa, but early signals of reduced in-vivo susceptibility to lumefantrine have been reported in Uganda [10]. This highlights the urgent need for continuous monitoring of drug levels and efficacy of artemether-lumefantrine to detect potential drug resistance development. Malaria is most prevalent in rural areas with limited infrastructure, making drug efficacy studies challenging. The DBS method offers a practical solution for field settings where reliable electricity delivery for freezers and equipment are lacking. The DBS technique is minimally invasive, requiring only a small blood sample from a finger or heel prick, which is then dried on filter paper. The sample can be stored and transported at room temperature in moisture-proof bags with desiccants, making it ideal for resource-limited environments.

However, artemether and its metabolite dihydroartemisinin cannot be quantified using the DBS method due to rapid degradation when blood hemolyzes on the filter paper, releasing reactive iron (Fe^2^⁺) [11]. This leaves lumefantrine as the primary target for DBS quantification, but developing a stable and reliable method for lumefantrine in DBS have been challenging.

Few studies have attempted to quantify lumefantrine in DBS. Early approaches tried stabilizing the drug using tartaric acid to pre-treat the filter paper [12], or adding phosphoric acid to the blood before spotting [13]. These methods successfully stabilized lumefantrine in DBS. However, both used liquid chromatography with ultraviolet detection (LC-UV), with lower limits of quantification (LLOQ) of 130 ng/ml and 160 ng/ml, respectively, barely sufficient to capture the expected day 7 lumefantrine concentrations. Other studies used plain filter paper but did not adequately address lumefantrine degradation. A recent study using LC-diode array detector reported a LLOQ of 500 ng/ml, which is too high to support measurement of Lumefantrine concentrations in clinical applications [14]. With LC–MS, lower quantification limits are possible. Gallay et al. developed a DBS method for seven antimalarial drugs, including lumefantrine, achieving a quantification limit of 20 ng/ml using pre-hemolyzed blood on FTA DMPK-B filter paper (Flinders Technology Associates “FTA” Drug Metabolism Pharmacokinetic “DMPK” Coating-B), which lyses cells and denatures proteins. However, after initial drying, they stored the DBS in freezers until analysis making it less suitable for field trials in resource-limited environments. The total run time was 12 min, which could be reduced to 7 min when analyzing only lumefantrine [9]. Ippolito et al. used plain filter paper but also stored the DBS frozen and transported it at ambient temperature. However, their method showed considerable validation variation, limiting it to semi-quantitative results [15].

The goal of a DBS method for lumefantrine is to enable blood sampling in remote, resource-limited areas, where blood is stored and transported at ambient temperature. Given the challenges in previous studies, we aimed to improve the pre-treated filter paper method [12], by a combination of improved sensitivity, shorter run time and better field suitability.

## Materials and methods

### Chemicals and reagents

Lumefantrine reference standard (purity 99.9%) and artemether (a concomitant drug, purity 100%) were obtained from AlsaChim (Illkirch, France). The stable isotope-labeled internal standard (SIL-IS), lumefantrine-d9 (purity 99.8%), was obtained from Toronto Research Chemicals (Toronto, ON, Canada). L-( +)-Tartaric acid ACS was obtained from Thermo Scientific Chemicals (MA, USA). Methanol, acetic acid, and ammonium formate, all MS-grade, were purchased from Honeywell (Seelze, Germany). Acetonitrile and formic acid, both MS-grade, were obtained from Fisher Chemicals (NJ, USA). Ultra-pure water (Type 1) was produced by a Millipore Direct-Q system from Merck (Darmstadt, Germany). Blank blood with heparin as anticoagulant was collected from healthy volunteers at the Faculty of Tropical Medicine, Mahidol University, Thailand (ethical approval: MUTM 2020-068-04). Chromatographic filter paper, Whatman 31 ET Chr, was obtained from GE Healthcare Life Sciences (Buckinghamshire, UK).

### Preparation of pre-treated filter paper

**Fig. 1:**
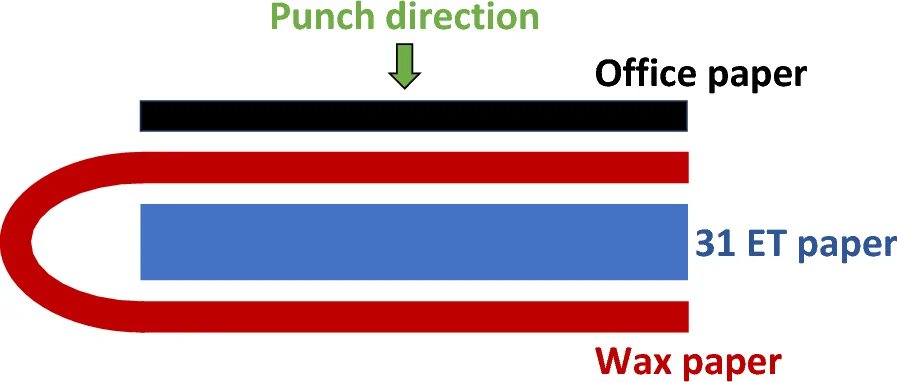
Layering of lubrication and cleaning paper for use in semi-automated punching robots

Initially, significant effort was dedicated to developing the lumefantrine DBS method using standard plain 31 ET Chr paper. However, lumefantrine extraction decreased rapidly during the drying process and continued to decrease even in dry storage, making the plain filter paper approach unsuitable. A possible workaround for the plain filter paper approach could be to rapidly degrade lumefantrine and then measure the degradation product. After extensive testing, including the use of oxidation and reduction agents, and other approaches including testing several different anticoagulants and DBS cards, it became clear that improving the recovery of lumefantrine in DBS on plain paper or using the degradation approach was not feasible. As a result, the focus shifted to redeveloping the previously published lumefantrine DBS method using treated paper [12]. However, the original method relied on manually cutting out the entire spot with scissors and then subdividing it with tweezers, which is time-consuming. Implementing an automated punching robot would improve sample throughput significantly. Unfortunately, the combination of the paper treatment and blood made the DBS samples sticky, frequently causing the punching robot to jam. A solution was found by lubricating the puncher with a wax surface, achieved by punching wax paper before and between the DBS samples. For semiautomated punching robots, this modification can be incorporated into the method, and the layering for both lubrication and punch cleaning can be seen in Fig. 1. The preparation of a layered paper stack using A4 paper (office printing paper), wax paper, and 31 ET paper, which can be secured with tape or staples, was implemented. However, waxed paper can become static and stick to the puncher. To prevent this, the A4 paper is placed on top, facing up, to help block the static waxed paper from attaching to the puncher. The pre-treated filter papers should be handled with care, as they can be irritating to bare skin. Additionally, the paper becomes more sensitive to repeated bending and may tear more easily than untreated paper, but this is only an issue if the DBS are sent in thin envelopes and experience rough handling.

Paper Pre-treatment: A solution of 0.75 mol/l tartaric acid in water was prepared and poured into a square plastic pan. The filter paper was soaked for about 4–5 s and then dried in a horizontal position on a wire net overnight. The treated papers were stored dry and protected from light until use.

### Preparation of stock solutions, calibrators and quality controls

Stock solutions of lumefantrine and SIL-IS were prepared in amber glass vials at a concentration of 1 mg/ml in their free forms using a methanol-acetic acid mixture (99.8:0.2 v/v). Two separate stock solutions of lumefantrine were prepared: one for the calibrators and one for the quality control (QC) preparation. Working solutions were prepared by diluting the stock solution with methanol-acetic acid (99.8:0.2 v/v). Calibrators and QCs were prepared by adding the working solution to blank blood to achieve a calibration range of 15–15,000 ng/ml. QC concentrations of 15, 45.1, 6803, 12,500, and 15,000 ng/ml, ranging from LLOQ to the upper limit of quantification (ULOQ), were prepared. An over-curve sample of 25,000 ng/ml was also prepared to test dilution integrity. The concomitant drug artemether was prepared in acetonitrile at a concentration of 1 mg/ml in its free form and added to some of the prepared lumefantrine spiked blood samples to a final concentration of 500 ng/ml to test the effects of the concomitant drug. Dried blood spots were prepared by adding 50 µl of blood to pre-treated filter paper, which was then dried at ambient room temperature overnight and transferred to a dry cabinet (set to 20% relative humidity) for long-term storage.

### LC–MS/MS

The LC system consisted of a Dionex Ultimate 3000 system with a binary LC pump, a vacuum degasser, a temperature-controlled well plate autosampler set to 6 °C, and a temperature-controlled column compartment set to 40 °C (Thermo Scientific, MA, USA). Data acquisition and processing were performed using Analyst 1.7 (Sciex, MA, USA). The LC column was a Luna-CN 50 × 2 mm, 3 µm, with a pre-column CN AJO-4304, 4 × 2 mm (Phenomenex, CA, USA), operated at a flow rate of 400 µl/min. The mobile phase consisted of acetonitrile-ammonium formate buffer (10 mM with 0.5% formic acid) in a 67:33 (v/v) ratio, with separation under isocratic conditions. The total runtime was 2 min per sample, with a 2 µl injection volume.

An atmospheric pressure ionization (API 5000) triple quadrupole mass spectrometer (Sciex, MA, USA) with a TurboV ionization source interface was used for the MS/MS analysis and operating in positive ion mode. Ion spray voltage was set to 5000 V, with a drying temperature at 650 °C. Curtain gas was set to 35 psi, and both nebulizer and auxiliary gases were set to 50 psi. The declustering potential was 100 V, and the entrance potential was 10 V. Quantification was performed using selected reaction monitoring for the specific transitions of lumefantrine: quantifier m/z 528.2 → 346.1 and qualifier m/z 528.2 → 276.1; for SIL-IS: quantifier m/z 537.2 → 346.1 and qualifier m/z 537.2 → 276.1. The collision energy and cell exit potential for quantifier were 55 V and 25 V, respectively, and for qualifier 85 V and 20 V. Data were processed using Analyst 1.7 (Sciex, MA, USA).

### Extraction method

Sample extraction was performed in a 96-well plate format. A semi-automated robotic punch instrument BSD600-Duet (Queensland, Australia) was used to punch out three discs of 3.2 mm in diameter from a single DBS (equivalent to about 10 μl of whole blood) into a 96-wellplate. Then, 300 µl of extraction solution (acetonitrile:water:acetic acid 75:25:1 v/v/v) containing 40 ng/ml SIL-IS was added. The sample plate was mixed on a Mixmate™ (Eppendorf, Hamburg, Germany) at 1000 rpm for 30 min. The extracted samples were then loaded on a HybridSPE®-PLus phospholipid removal plate (Supelco, Sigma-Aldrich, MO, USA) and filtrated through using 8-inch Hg vacuum pressure, with eluates collected in a new 96-well plate. The sample plate with the eluates was mixed on a Mixmate™ at 500 rpm for 3 min before being placed in the autosampler.

### Method validation

Method validation followed the guidelines of the International Council for Harmonization (ICH M10, 2022) and the U.S. Food and Drug Administration (FDA, 2018) [16, 17], where the acceptance criteria for precision and accuracy are ± 15%, except for LLOQ, where ± 20% is accepted.

#### Selectivity and specificity

Blank human DBS from six different sources of healthy volunteers were extracted and evaluated. Additionally, one of the blood sources was lipemic. Acceptance criteria required that none of the sources should produce a signal exceeding 20% of the standard at the LLOQ and no more than 5% of the internal standard response.

One concomitant drug, artemether and a metabolite of lumefantrine, desbutyl-lumefantrine along with several possibly co-administered drugs, ivermectin (antiparasitic drug), HIV drugs efavirenz, ritonavir, lopinavir, and paracetamol (acetaminophen), were injected in neat solution to investigate any signal response at the retention time of lumefantrine that would indicate an interference.

#### Recovery and matrix effects

Recovery (absolute recovery) was evaluated at three concentration levels: 45.1, 6,803 and 12,500 ng/ml. The first set of samples (set-1) consisted of five replicates per concentration of pre-fortified DBS samples, extracted with SIL-IS as described in extraction method above.

The second set of samples (set 2) consisted of extracted blank DBS samples from the six different blank donor sources, including the lipemic DBS donor, and then post-extraction fortified with lumefantrine and SIL-IS to achieve the same nominal concentration as the first set. Recovery was determined by comparing the peak response of individual pre-fortified samples (set-1) to the average peak response of post-extract fortified samples (set-2).

For matrix effect evaluation, a third set of samples (set-3) was prepared, consisting of five replicates of neat solution at the same nominal concentration as above (45.1, 6,803 and 12,500 ng/ml). Matrix effects were evaluated using a simplified approach described by Matuszewski et al. [18]. Matrix effects were calculated by comparing the average peak response of post-extract fortified samples of the second set of samples to the average response of the neat solution from the third set of samples at each concentration level evaluated.

#### Precision and accuracy and linearity

Linearity was evaluated by analyzing the calibration standards from four separate runs, with each calibration point run as two replicate samples. Linear and quadratic regression models were evaluated and the best performing model was selected based on the lowest total sum of relative residuals of back-calculated concentrations compared to nominal concentrations. Precision and accuracy were evaluated using five replicates of each QC level over four analytical runs. A total of five QC levels, ranging from LLOQ (15 ng/ml) to ULOQ (15,000 ng/ml), were evaluated.

#### Carry-over

The carry-over effect was investigated by injecting five ULOQ samples (15,000 ng/ml), followed by three replicates of blank samples. This was evaluated on three different days during the validation. A signal was considered significant if it exceeded 20% of the LLOQ or 5% of the IS response at the respective retention times.

#### Dilution integrity

Dilution integrity was evaluated using five replicates of over-curve samples at a concentration of 25,000 ng/ml. The over-curve samples were diluted to fall within the calibration range by punching one disc of the over-curve DBS sample, followed by two punches of a blank DBS sample, resulting in a three-fold dilution factor. The mean accuracy should be within ± 15% of the nominal concentration and precision should not exceed 15% relative standard deviation.

#### Re-injection reproducibility

Re-injection reproducibility was tested by re-injecting a full set of calibration and QC samples that had been stored in the autosampler at 6 °C for 44 h.

#### Stability

Stock solution (1 mg/ml) and the lowest working solution (660 ng/ml) was evaluated at 22 °C for 6 h, and in 6 °C and -80 °C for 17 days. All stability evaluations in DBS samples were performed at concentrations of 45.1, and 12,500 ng/ml. Freeze/thaw stability was evaluated up to 5 cycles where DBS samples were frozen to -80 °C and thawed to room temperature for 2–3 h before being returned to the freezer. Benchtop sample process stability was assessed by adding the extraction solvent and leaving the samples at room temperature for up to 4 h before continuing with the extraction procedure.

To better understand the optimal drying time for blood spots before use, a short-term sample drying stability study was conducted. During sample preparation and blood spotting, QC sets were allowed to dry for different durations (24, 48 h, 7, 14, and 29 days). The initial 24 h of drying were conducted in a laboratory air-conditioned room (22–25 °C, 40–50% RH), after which the samples were stored in a dry cabinet (set to 20% RH) until transfer to -80 °C storage. All time points were compared to a QC set dried for 4 h and stored at -80 °C. This study was performed using both pre-treated and non-treated filter paper. Long-term stability was evaluated after 4 months and also included samples containing the concomitant drug artemether. Another set, prepared and stored dark and dry for 16 years, was also included. These quality control samples had been prepared in another research unit as described in a previous publication [12], and later transferred to our laboratory for testing. Five replicate DBS were analyzed per concentration. The low and high concentration was 212 and 5290 ng/ml, respectively. A calibration curve was freshly prepared and stored for 4 months until used for the quantification of the 16-year-old DBS.

#### Interference between analyte and internal standard SIL-IS

For interference testing between lumefantrine and the SIL-IS, the first set of samples (set-A) consisted of an injection of extraction solvent containing only the internal standard to investigate any signal interference from SIL-IS to lumefantrine. The second set of samples (set-B) involved the extraction of ULOQ DBS samples without SIL-IS to evaluate if lumefantrine at ULOQ level would cause interference with SIL-IS. The third set of samples (set-C) involved the extraction of LLOQ DBS samples with the internal standard as a reference for the signal intensity of both compounds to quantify the level of interference. All sample sets were done in triplicates. The presence of a signal greater than 20% of the LLOQ or 5% of the internal standard indicates interference.

#### Variations due to DBS punch position (DBS spot homogeneity)

To test if the dried sample spots were homogenous throughout the surface area, sample punches were made from both the center and the edge of the DBS. The center punches would be the reference point, and the concentration difference of the edge punches were calculated.

#### Hematocrit effects

Whole blood was centrifuged at 2000×g for 10 min, after which plasma was added to obtain an erythrocyte volume fraction of 20%. The blood was mixed and then fortified with lumefantrine to a concentration of 45.1 and 12,500 ng/ml and subsequently spotted onto pre-treated filter paper. Measurements from the low-hematocrit samples were compared with a calibration curve generated from blood spots obtained from healthy volunteers (40% hematocrit). Five replicate samples at each concentration were analyzed.

## Result and discussion

A comparison of newly treated paper and 6-month-old treated paper showed no difference in paper strength or tear sensitivity over time. Furthermore, the quantification result, as both papers were used in the validation, were indistinguishable between the two pre-treatment dates. A key precaution is that lumefantrine, when in neat solutions such as stock solutions or working solutions, has a tendency to adsorb to plastic surfaces. Therefore, all lumefantrine solutions should be prepared in glass tubes or vials [19].

### Method validation

#### Selectivity and specificity

Analysis of six blank DBS samples from healthy volunteers—including one lipemic sample—showed adequate separation and that no endogenous interference peaks at the retention time of lumefantrine (supplement Figure S1). For specificity, injections of neat solutions of the concomitant drug artemether and the metabolite of lumefantrine, desbutyl-lumefantrine, as well as several possibly co-administered drugs (ivermectin, efavirenz, ritonavir, lopinavir and paracetamol), exhibited no interference with lumefantrine (supplement Figure S2).

#### Recovery and matrix effects

Absolute recovery of lumefantrine was in the range of 38–46% and was independent of the concentration level (Table 1). This is lower than the previously published method (60–65%) [12] and slightly lower than phosphoric acid fortified blood DBS method (45–51%) [13], but higher than the untreated method (10–38%) [15]. One reason for the lower recovery in the present method is that this DBS method only extracts the DBS for 30 min for fast sample processing and the DBS had already been stored for several weeks when the recovery test was performed. It takes at least a week for the DBS to equilibrate and more closely represent real clinical study samples. A deeper discussion on stability is presented in Sect. "Stability" below. The SIL-IS recovery was 97–99%, which is expected since the SIL-IS is added in solution in the extraction process and is not dried as lumefantrine is in the DBS.

**Table 1:** Recovery and matrix effect of lumefantrine and SIL-IS in DBS

Sample	Nominal (ng/ml)	Recovery (%)	RSD (%)	Matrix effect (%)	RSD (%)
Lumefantrine QC1	45.1	45.6	8.4	89.2	2.0
Lumefantrine QC2	6,803	38.1	6.9	90.4	2.1
Lumefantrine QC3	12,500	44.4	1.7	92.9	2.5
SIL-IS for QC1	40	97.9	4.0	88.6	1.2
SIL-IS for QC2	40	98.8	1.3	91.2	1.3
SIL-IS for QC3	40	97.1	1.3	92.4	2.9

Recovery: average absolute recovery of five individual QC samples, calculated as the peak response of individual pre-fortified samples (set-1) compared to the average peak response of post-extract fortified samples (set-2), recovery does not have a pass/fail criteria. Matrix effect: average matrix effect of six individual donors, calculated as the peak response of an individual blank post extract fortified sample (set 3) compared to the average response of post-extract fortified samples (set-2), matrix effects should stay within 100 ± 15%. RSD: relative standard deviation, calculated as standard deviation divided with the mean value, RSD should be within ± 15%

None of the six donors, including the lipemic donor sample, showed any matrix effects for lumefantrine or the SIL-IS (Table 1). The matrix effect calculation should be at 100 ± 15%.

#### Precision, accuracy and linearity

The simplest regression model, which provided the best predictions for calibrators and QC samples, was a linear regression model with 1/x weighting. All competing models resulted in inferior predictive performance.

All precision and accuracy tests met the validation criteria, with precision below 13% at all concentration levels, and accuracy ranging from 100–114.5% indicating a slight bias in the preparation between calibrators and QC samples (Table 2).

**Table 2:** Accuracy and precision of lumefantrine in DBS

Sample	Nominal (ng/ml)	Precision (%)		Accuracy (%)
		Intra-assay	Inter-assay	
LLOQ	15.0	5.77	12.2	107.5
QC1	45.1	5.06	10.7	114.2
QC2	6,803	7.22	12.0	100.5
QC3	12,500	6.24	8.71	103.0
ULOQ	15,000	5.61	3.55	114.5

Acceptance criteria: Precision intra- and inter-assay should be within ± 15%. Accuracy should stay within 100 ± 15%

#### Carry-over

**Fig. 2:**
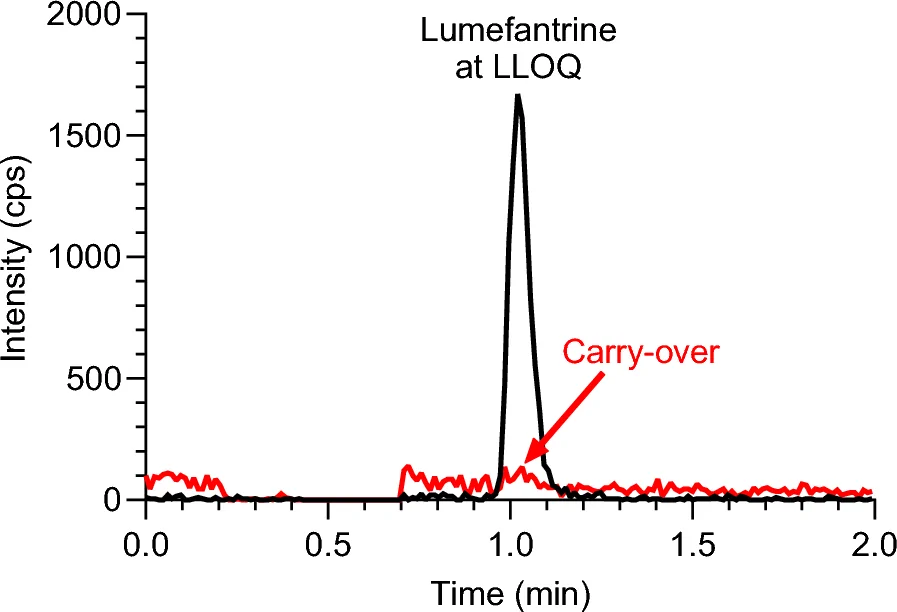
Selectivity. A lumefantrine-extracted DBS sample at LLOQ (15 ng/ml) was overlaid with the first blank injection response after injecting five ULOQ samples. Carryover should not exceed 20% of the LLOQ. The absence of signal between 0.2 and 0.7 min is caused by a switch valve, diverting the mobile phase flow to waste, reducing the risk of contamination from early-eluting components

Potential carry-over effects were evaluated by injecting three extracted blank samples immediately after five replicate injections of the highest concentration in the calibration curve. SIL-IS was completely free from carry-over, while lumefantrine exhibited a small tendency of a carry-over (Fig. 2). However, all peaks were well within the carry-over limit of 20% of the LLOQ, hence there was no significant carry-over.

#### Dilution integrity

Five replicates of over-curve DBS concentrations (25,000 ng/ml) were diluted threefold with blank DBS spots (1 over-curve DBS punch + 2 blank DBS punches) and injected to assess dilution integrity. The dilution integrity test achieved an intra-assay precision of 6% with an average accuracy of 110% upon back-calculation to the original concentration.

#### Re-injection reproducibility

Reinjection reproducibility in the LC-autosampler at 6 °C was evaluated, where the entire set of calibrators and QCs was re-injected after 44 h. The reinjection comparison of low and high QC 45.1 and 12,500 ng/ml showed not more than a 2.1% difference between the original and re-injected concentrations. Precision was within 9% variation, demonstrating good reproducibility for up to 44 h in the event of instrument failure or other interruptions.

#### Stability

Stock solution (1 mg/ml) and the lowest working solution at (660 ng/ml) were evaluated at 22 °C for 6 h and in 6 °C and -80 °C for 17 days. The stock solution was stable with 97–99% accuracy and the working solution stayed within 102–104% accuracy.

Stability in DBS was evaluated at concentrations of 45.1, and 12,500 ng/ml. Freeze/thaw stability was good for up to 5 cycles, with accuracies of 95–114% for lumefantrine alone and 92–106% in the presence of the concomitant drug artemether.

Benchtop sample process stability, where extraction solvent was added to the DBS and left at room temperature for 4 h before continuing with the extraction procedure, showed increasing absolute recovery over time. After two hours, the recovery had increased with 50% and after 4 h the recovery was 63–85% higher. During method development, extraction times of 20, 40, 60 and 90 min were evaluated. While 20–60 min gave similar extraction recovery, the 90-min extraction showed 23% higher recovery. However, one must also consider the time efficiency in the laboratory, as leaving samples to extract for 4 h is inconvenient. A 30-min extraction was chosen as a suitable compromise between time and efficiency, as extraction recoveries were similar across the 20–60 min range. Since calibrators and QCs are extracted alongside the samples, any variations in extraction affected all samples equally.

**Fig. 3:**
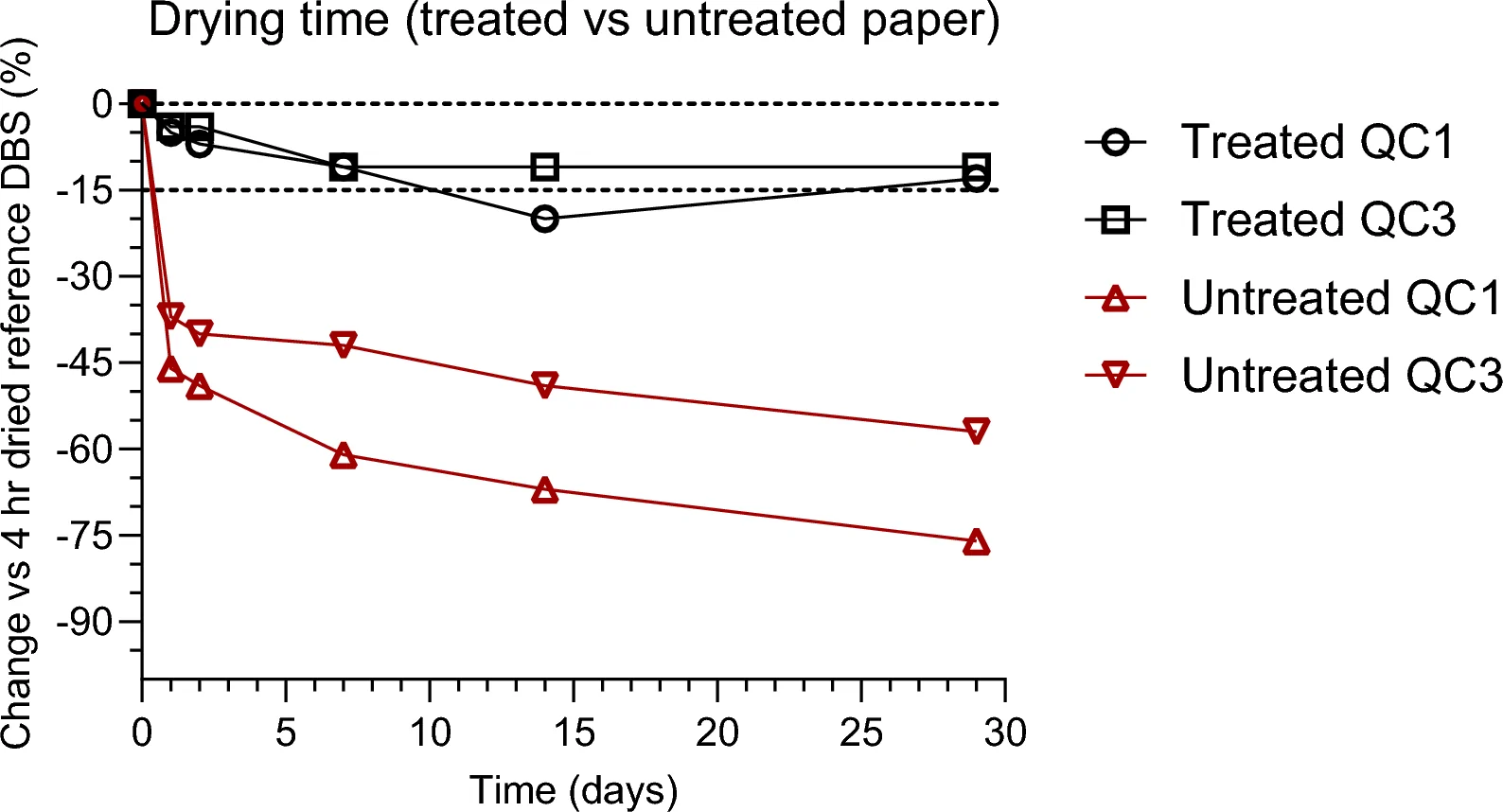
Drying and extraction efficiency profiles of pre-treated and untreated paper compared to a reference set dried for only 4 h. After the pre-determined drying time, DBS spots were transferred and stored in a − 80 °C freezer until analysis

Drying stability tests showed significant differences in extraction efficiency depending on how long DBS spots had dried. For treated paper, the change occurred mainly in the first 7 days, where the extraction efficiency dropped by about 10–15%. Both high and low concentration showed similar trends with no significant difference (Fig. 3). In the previous publication, a few more timepoints were used (7, 28, 49, 70 and 120 days) and it showed that after 7 days of drying, the recovery had equilibrated and there was no additional change in the following time points. That study also had a follow-up timepoint of 2.5 years (unpublished) that showed no change for DBS on pre-treated 31 ET Chr filter paper. For the analysis of study samples, calibrator and QC DBS prepared on pre-treated filter paper should be allowed to dry for at least 7 days to equilibrate and reach a stable state before being used for drug quantification. In this study, we also used a DBS calibrator set prepared on pre-treated filter paper and stored for 4 months to assess the stability of DBS samples that had been stored for 16 years on the same type of paper. The 16-year stored DBS samples showed accuracies of 108–110%, indicating no evidence of degradation during long-term storage over many years.

For comparison, lumefantrine DBS on untreated paper was also investigated, and the result were quite different. Extraction efficiency of lumefantrine on untreated paper dropped rapidly in the first two days with a 40–50% loss, continuing to decrease over time. After 1 month, the low QC1 concentration had lost 76%, while the high QC3 concentration had lost 57% (Fig. 3). A similar trend of reduced extraction recovery over time was also reported previously using untreated filter paper [15]. Hence, for untreated papers, there is a concentration-dependent reduction, where lower concentrations are reduced much faster than higher concentrations, which is problematic for methods trying to use untreated papers during sample collection. Unfortunately, there were no long-term stability samples available for untreated paper, but the 1-month test showed that untreated papers are unsuitable as a sample matrix for lumefantrine blood collection and storage.

Additionally, DBS stability samples on pre-treated filter paper, with or without the presence of a concomitant drug showed that lumefantrine alone had an accuracy of 97–104%, while lumefantrine in the presence of artemether showed an accuracy of 96–109%. These results indicate comparable performance and no adverse impact from the concomitant drug.

#### Interference between analyte and SIL-IS

**Fig. 4:**
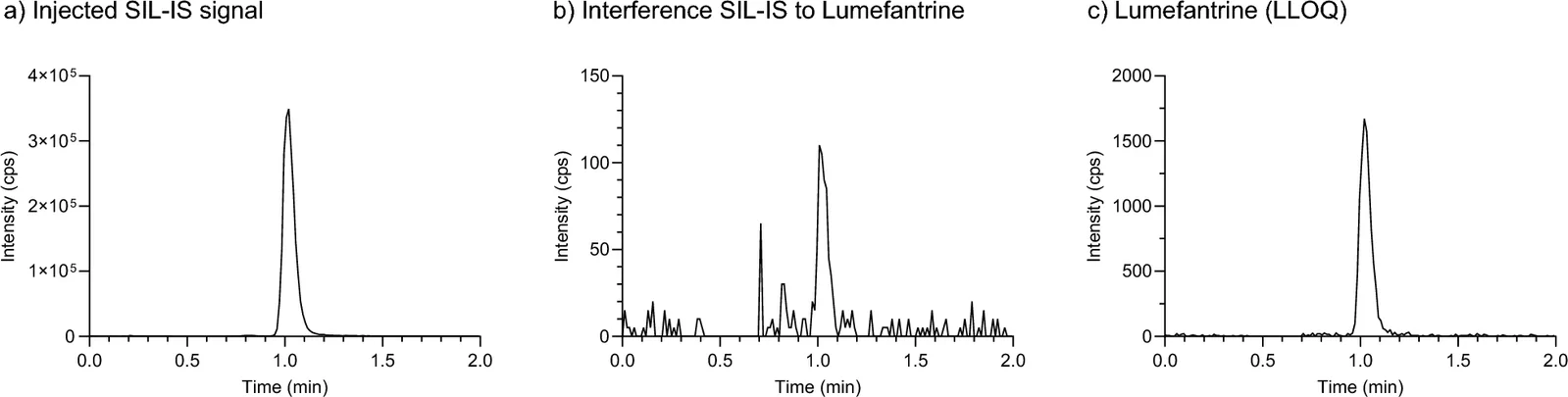
Interference between lumefantrine and SIL-IS. The graphs show signal intensity of the injected SIL-IS extraction solvent (**a**), a small visible cross-interference with lumefantrine, corresponding to approximately 8% of the LLOQ signal (**b**), and lumefantrine signal intensity at LLOQ for comparison (**c**)

No significant signal interference was detected between SIL-IS and lumefantrine when injecting the extraction solvent containing SIL-IS. Only a small peak was visible, accounting for 8% of lumefantrine at LLOQ, well below the 20% of LLOQ validation cutoff limit. Injecting an extracted lumefantrine ULOQ sample without SIL-IS showed no signal response for the SIL-IS, hence, no interference between lumefantrine at ULOQ and SIL-IS (Fig. 4).

#### Variations due to DBS punch position (DBS spot homogeneity)

**Fig. 5:**
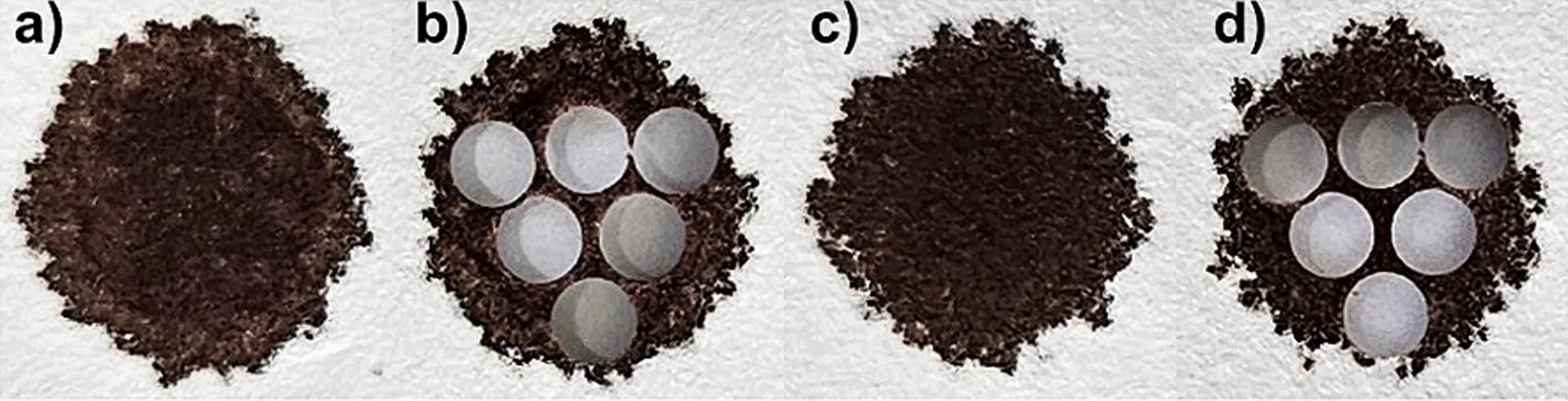
DBS punching homogeneity on pre-treated filter paper. Front side of the DBS (**a**), punched front side of the DBS (**b**), back side of the DBS (**c**), and punched back side of the DBS (**d**)

To assess whether the DBS spots were homogeneous across the surface area, three punches were taken from the center of the DBS spot, followed by three punches from the edge of the same spot to evaluate the possibility of re-punching the same DBS spot. Visually, the DBS spot on the treated filter paper appeared darker in the center and lighter near the edge (Fig. 5). This gradient was only noticeable on the blood application side, not on the reverse side of the DBS. Comparison of the quantification results between the center and edge punches showed some variation, with the highest deviation being ± 9% (n = 5), which falls within the ± 15% analytical acceptance criteria. These findings indicate that punches taken from either the center or closer to the edge yield comparable quantification results.

#### Hematocrit effects

Malaria infection leads to hemolysis of infected erythrocytes, and as the disease progresses, hematocrit levels decrease [20]. Hematocrit can influence blood spot size, as blood with a lower hematocrit might diffuse more readily across filter paper, producing larger spots. Consequently, when using a fixed-area punch, less blood is collected per sample.

Quantification of 20% hematocrit DBS using calibration sample set prepared from 40% hematocrit blood resulted in accuracies of 92% for the low concentration (45.1 ng/ml) and 95% for the high concentration (12,500 ng/ml). This indicates a slight underestimation at lower hematocrit levels, but the results remain well within the acceptable method variation of 100% ± 15%.

## Conclusions

Using pre-treated filter paper is the optimal approach, as it addresses many of the issues encountered with plain filter paper. This method facilitates clinical studies on lumefantrine treatment in resource-limited settings, where facilities might be lacking, and the small blood volume required (50 µl) makes capillary blood collection a feasible sampling technique in under-served populations such as young children. To achieve the most reliable quantification results, prepared DBS calibrators and QC samples should be allowed to dry and be stored in a dry environment for at least one week, preferably longer, to ensure that recovery is consistent with that of the study samples. As our results demonstrate, 16-year-old lumefantrine DBS on treated filter paper and 4-month-old preparations showed nearly identical recovery, indicating excellent stability over a long period of time. The described method is sufficiently sensitive to reliably quantify lumefantrine concentrations at day 7 and may also be applicable for quantifying day 28 samples, which are sometimes used to assess drug concentrations in cases of recurrent malaria. Given the specific precautions regarding adsorption, handling of pre-treated paper, and drying profiles outlined in this paper, we present a reliable quantification method using modern instrumentation and sample processing techniques.

## Supplementary Information

Additional file1 (DOCX 344 KB)

## Data Availability

Data will be made available on request.
