# Supplementary material for: Lumefantrine quantification on pre-treated filter paper dried blood spots for clinical field trials
Source: Malar J. 2026 Jul 20;25:366. doi: 10.1186/s12936-026-06048-y (PMC13663207; doi:10.1186/s12936-026-06048-y)
Supplement: Supplementary file 1 [file 12936_2026_6048_MOESM1_ESM.docx]

**Supplementary material**

**Lumefantrine Quantification on Pre-Treated Filter Paper Dried Blood Spots for Clinical Field Trials**

*Daniel Blessborn^1 2^, Natpapat Kaewkhao^1^, Joel Tarning^1 2^*

^1^ Mahidol Oxford Tropical Medicine Research Unit, Faculty of Tropical Medicine, Mahidol University, Bangkok, Thailand**.** ^2^ Centre for Tropical Medicine & Global Health, Nuffield Department of Clinical Medicine, University of Oxford, Oxford, UK

The Supplementary Materials include:

- **Figure S1.** Selectivity test analysis of six blank DBS samples, from six healthy volunteers (Donor A-F). Blank water and lumefantrine LLOQ sample are also visualized for signal reference.
- **Figure S2.** Specificity test. Injections of neat solutions of lumefantrine at LLOQ (a), the metabolite of lumefantrine, desbutyl-lumefantrine (b), the concomitant drug artemether (c), and several possibly co-administered drugs (d-h).

**Figure S1**. Selectivity test analysis of six blank DBS samples, from six healthy volunteers (Donor A-F). Blank water and lumefantrine LLOQ sample are also visualized for signal reference.

**Figure S2**. Specificity test. Injections of neat solutions of lumefantrine at LLOQ (a), the metabolite of lumefantrine, desbutyl-lumefantrine (b), the concomitant drug artemether (c), and several possibly co-administered drugs (d-h).
